# Inducible and Acquired Clarithromycin Resistance in the *Mycobacterium abscessus* Complex

**DOI:** 10.1371/journal.pone.0140166

**Published:** 2015-10-08

**Authors:** Marc Rubio, Francesca March, Montserrat Garrigó, Carmen Moreno, Montserrat Español, Pere Coll

**Affiliations:** 1 Departament de Genètica i Microbiologia, Universitat Autònoma de Barcelona, Edifici C 08193, Bellaterra (Cerdanyola del Vallès), Spain; 2 Servei de Microbiologia, Fundació de Gestió de l’Hospital de la Santa Creu i Sant Pau, C/Sant Quintí 89, 08026, Barcelona, Spain; Cambridge University, UNITED KINGDOM

## Abstract

**Purpose:**

Clarithromycin was considered the cornerstone for the treatment of *Mycobacterium abscessus* complex infections. Genetic resistance mechanisms have been described and many experts propose amikacin as an alternative. Nevertheless, clarithromycin has several advantages; therefore, it is necessary to identify the non-functional *erm(41)* allele to determine the most suitable treatment. The aims of this study were to characterize the molecular mechanisms of clarithromycin resistance in a collection of *Mycobacterium abscessus* complex isolates and to verify the relationship between these mechanisms and the antibiogram.

**Materials and Methods:**

Clinical isolates of *M*. *abscessus* complex (n = 22) from 16 patients were identified using four housekeeping genes (*rpoB*, *secA1*, *sodA* and *hsp65*), and their genetic resistance was characterized by studying *erm(41)* and *rrl* genes. Nine strains were recovered from the clinical isolates and subjected to E-test and microdilution clarithromycin susceptibility tests, with readings at 3, 7 and 14 days.

**Results:**

We classified 11/16 (68.8%) *M*. *abscessus* subsp. *abscessus*, 4/16 (25.0%) *M*. *abscessus* subsp. *bolletii*, and 1/16 (6.3%) *M*. *abscessus* subsp. *massiliense*. T28 *erm(41)* allele was observed in 8 *Mycobacterium abscessus* subps. *abscessus* and 3 *Mycobacterium abscessus* subsp. *bolletii*. One strain of *M*. *abscessus* subsp. *bolletii* had an *erm(41)* gene truncated and was susceptible to clarithromycin. No mutations were observed in *rrl* gene first isolates. In three patients, follow-up of initial *rrl* wild-type strains showed acquired resistance.

**Conclusions:**

Most clinical isolates of *M*. *abscessus* complex had inducible resistance to clarithromycin and total absence of constitutive resistance. Our findings showed that the acquisition of resistance mutations in *rrl* gene was associated with functional and non-functional *erm(41)* gene. Caution is needed when using *erm(41)* sequencing alone to identify *M*. *abscessus* subspecies. This study reports an acquired mutation at position 2057 of *rrl* gene, conferring medium-low clarithromycin constitutive resistance.

## Introduction

*Mycobacterium abscessus* complex subspecies are rapid-growing mycobacteria (RGM) responsible for chronic pulmonary infections, cutaneous infections and, in some cases, bacteraemia [1,2]. These subspecies are considered as the most drug-resistant of all the RGM [3–5], which complicates treatment of the infections they cause. Macrolides such as clarithromycin and azithromycin were considered the cornerstone for the treatment of *Mycobacterium abscessus* complex infections [5]. Molecular resistance mechanisms have been described and many experts propose amikacin as an alternative [6]. Nevertheless, clarithromycin has several advantages; therefore, it is necessary to identify the non-functional *erm(41)* allele to determine the most suitable treatment.

Clarithromycin resistance in the *M*. *abscessus* complex can be constitutive or inducible, depending on two mechanisms. Constitutive resistance involves spontaneous point mutations, selected during macrolide-based chemotherapy, at positions 2058 and 2059 of the *rrl* gene, which encodes for the 23S rRNA [7]. The second mechanism is associated with inducible resistance [8], involving the presence of a functional *erm(41)* gene and following methylation of the ribosome. Constitutive resistance is characterized by *in vitro* high level resistance values to clarithromycin at 3 days of culture [4,7], while inducible resistance associated with the *erm(41)* gene is characterized by *in vitro* MIC resistant values only at 7 and even 14 days [9].

The taxonomy of the *M*. *abscessus* group is complex and still unresolved. In 2011, *M*. *bolletii* and *M*. *massiliense* were classified as a single subspecies (*M*. *abscessus* subsp. *bolletii*) due to their low genetic diversity [10]. In 2013, whole genome sequencing data analysis supported the differentiation of the *M*. *abscessus* complex into 3 subspecies: *M*. *abscessus* subsp. *abscessus*, *M*. *abscessus* subsp. *bolletii* and *M*. *abscessus* subsp. *massiliense* [11]. In the present study, we use this nomenclature for the three subspecies.

It is widely accepted that several housekeeping genes are needed to identify the three subspecies in the complex [12,13]. These three subspecies are closely related, and recombination and gene transfer has been described [11]. The genes most commonly used to classify *M*. *abscessus* subspecies are the r*poB*, *secA1*, *sodA*, *recA* and *hsp65* genes [12,13]. Even when powerful new techniques such as matrix-assisted laser desorption/ionization time-of-flight (MALDI-TOF) [14] are used, identification of *M*. *abscessus* complex isolates at the subspecies level is not 100% accurate.

The *erm(41)* gene differs slightly between *M*. *abscessus* complex subspecies. A complete *erm(41)* gene with 10 sequevars is described for *M*. *abscessus* subsp. *abscessus* [15]: sequevars with nucleotide T28 are associated with inducible clarithromycin resistance, and the ones with nucleotide C28 are linked to clarithromycin susceptibility. In *M*. *abscessus* subsp. *bolletii*, the *erm(41)* gene is similar to T28 *M*. *abscessus* subsp. *abscessus* [16]. In *M*. *abscessus* subsp. *massiliense*, the *erm(41)* gene is known to have 2 deletions, making it non-functional. Sequence analysis of the *erm(41)* gene has been used to classify *M*. *abscessus* complex subspecies [17].

We collected *M*. *abscessus* complex clinical isolates from 1995 to 2014 at a university hospital in Spain. The aims of this study were to characterize the molecular mechanisms of clarithromycin resistance in a collection of *Mycobacterium abscessus* complex initial isolates, identified at the subspecies level, and to verify the relationship between the genetic resistance mechanisms and the antibiogram results. We also studied 10 isolates in the follow-up of 4 patients by molecular typing and phenotypic and genotypic antibiograms.

## Materials and Methods

### Ethics statement

This study was retrospective without interaction with patients and all the patients’ information was de-identified prior to analysis. Therefore the Fundació de Gestió Sanitaria del Hospital de la Santa Creu i Sant Pau ethics committees waived the need for informed consent and approved this research study (IISBP-CLA-2014-23).

### Samples

Twenty-two clinical isolates were obtained between 1995 and 2014 from 16 patients: 3 from skin and 19 from lung infections. Ten isolates from 4 patients were used for follow-up: 3 from patient 1, 3 from patient 2, 2 from patient 3, and 2 from patient 4. DNA from all isolates was recovered for genotypic identification and molecular clarithromycin resistance analysis. Nine first recovered isolates from 9 individual patients were subjected to clarithromycin susceptibility tests.

### Subspecies classification

Total DNA was extracted from mycobacterial clinical isolates by thermal shock [18] or InstaGene matrix (Bio-Rad Laboratories, Hercules, CA, USA). Analysis of *rpoB*, *secA1*, *sodA* and *hsp65* genes was performed for identification at the subspecies level, as described previously [12,13]. PCR products were sent to Macrogen for sequencing (Meibergdeef, 1105AZ Amsterdam, The Netherlands). The sequences obtained were analyzed using the BLAST algorithm (NCBI).

### Assessing the genetic basis of resistance

The *erm(41)* and *rrl* genes were analysed for clarithromycin resistance. The *erm(41)* gene was amplified using primers ermF (5’-GACCGGGGCCTTCTTCGTGAT-3’) and ermR (5’-GACTTCCCCGCACCGATTCC-3’) [7,8]. The *rrl* gene was amplified using primers 19F (5’-GTAGCGAAATTCCTTGTCGG-3’) and 21R (5’-TTCCCGCTTAGATGCTTTCAG-3’) [7]. For the *erm(41)* gene, we analysed the complete gene sequence of 673 bp and T28 polymorphism. For the *rrl* gene, we analysed a fragment of 836 bp that included nucleotides 2058–2059, whose mutations cause clarithromycin resistance.

For *erm(41)* and *rrl* amplification, the PCR conditions were 5 min at 95°C, then 35 cycles at 95°C for 60 s, 62°C (for *erm(41)* but 55°C for *rrl*) for 60 s, and 72°C for 90 s, followed by 72°C for 10 min in the final extension.

### Reference sequences

The reference sequences used for subspecies classification were *M*. *abscessus* subsp. *abscessus* ATCC 19977T, *M*. *abscessus* subsp. *bolletii* reference strain CCUG 50184 (GenBank accession numbers AY859692 for *rpoB* and FJ442839.1 for *secA1*), *M*. *abscessus* subsp. *bolletii* reference strain CIP 108541 (GenBank accession numbers EU266576.1 for *hsp65* and AY862403 for *sodA*), and *Mycobacterium abscessus* subsp. *massiliense* CCUG 48898 (GenBank accession numbers AY593981 for *rpoB*, AY596465.1 for *hsp65*, NZ_AKVF01000005.1 for *secA1* and AY593975 for *sodA*).

The reference sequence used for the analysis of *erm(41)* and *rrl* genes was the *M*. *abscessus* subsp. *abscessus* ATCC 19977T (GenBank accession number NC_010397).

### Clarithromycin susceptibility testing

Clarithromycin MIC determination was performed in 9/16 initial isolates by the E-test method (bioMérieux, bioMérieux España S.A., Madrid, Spain) in Mueller-Hinton agar plates at 30°C [19]. The clarithromycin susceptibility test was also done in cation-adjusted Mueller-Hinton medium using the broth microdilution method [5] in Sensititre RAPMYCOI plates (Sensititre, Trek Diagnostic Systems, East Grinstead, United Kindom) according to CLSI guidelines [9]. Strains were incubated for 14 days, and clarithromycin MIC was read at 3, 7 and 14 days. Clarithromycin MIC cut-off for microdilution ranges from ≤2 susceptible to ≥8 resistant, and ≤1 susceptible to ≥4 resistant for the E-test [19].

### Patient follow-up

Strain typing in the patient follow-up was performed by variable-number of tandem-repeat analysis (VNTR). The primers used were TR45, TR109, TR116, TR150, TR155 and TR172. The six pairs of primers and loci were the same as used by Wong *et al*. [20]. The PCR conditions for VNTR were as follows: a initial denaturalization step of 15 min at 95°C; 30 cycles of denaturalization at 95°C for 60 s, annealing at 59°C for 60 s and an extension at 72°C for 90 s, with a final elongation step at 72°C for 10 min. PCR amplicons were analyzed using the EPOCH capillary electrophoresis system (BioTek, Germany). Results were analyzed using GeneMapper (Applied Biosystems).

### Sequences accession numbers

The obtained sequences for each gene were submitted to GenBank and given the following accession numbers: KT185536 to KT185557 for *rpoB*, KT185514 to KT185535 for *hsp65*, KT185603 to KT185624 for *sodA*, KT185581 to KT185602 for *secA1*, KT185492 to KT185513 for *erm41* and KT185558 to KT185580 for *rrl*.

## Results

### Multiple gene-based subspecies classification

Analysis of the initial isolate from each patient identified 11/16 *M*. *abscessus* subsp. *abscessus* (68.8%), 4/16 *M*. *abscessus* subsp. *bolletii* (25.0%) and 1/16 *M*. *abscessus* subsp. *massiliense* (6.3%). For all isolates except one, we observed agreement between *rpoB*, *secA1*, *sodA* and *hsp65* genes sequences. One strain (isolate 08I004 from Table 1) was *M*. *abscessus* subsp. *abscessus* according to *rpoB*, *sodA* and *hsp65* analysis but *M*. *abscessus* subsp. *bolletii* according to *secA1* gene. Table 1 shows subspecies classification of the 16 first clinical isolates included in the study.

**Table 1:** Clarithromycin resistance molecular mechanisms, clarithromycin susceptibility testing and subspecies identification of *M*. *abscessus* complex strains.

								MIC ^d^ ^,^ ^e^ ^,^ ^f^ ^,^ ^g^ ^,^ ^h^					
								Microdilution			E-test		
Patient	Strain	Date	Identification of the strain	VNTR profile ^a^	Source	*Erm* nt ^b^ 28 (Functionality)	*rrl* ^c^	3D	7D	14D	3D	7D	14D
1	07I011	05/06/2007	*M*. *abscessus* subsp. *abscessus*	Profile I	Lung	C (Not functional)	Wt	<0.06	0.25	<2	<0.016	0.19	1.5
1	10I281	24/10/2010	*M*. *abscessus* subsp. *abscessus*	Profile I	Lung	C (Not functional)	A2058G	>16			>256		
1	13I330	08/10/2013	*M*. *abscessus* subsp. *abscessus*	Profile I	Lung	C (Not functional)	A2058G	>16			>256		
2	09I152	24/03/2009	*M*. *abscessus* subsp. *abscessus*	Profile II	Lung	T (functional)	Wt	<0.06	4	>16	0.38	>256	>256
2	11I110	23/02/2011	*M*. *abscessus* subsp. *abscessus*	Profile II	Lung	T (functional)	Wt	<2			0.75		
2	11I441	12/12/2011	*M*. *abscessus* subsp. *abscessus*	Profile II	Lung	T (functional)	A2057G/A2057 ^i^	8			64		
3	09I380	17/09/2009	*M*. *abscessus* subsp. *abscessus*	Profile III	Lung	T (functional)	Wt	0.12	8	>16	0.094	4	>256
3	11I289	05/07/2011	*M*. *abscessus* subsp. *abscessus*	Profile III	Lung	T (functional)	A2059G	>16			>256		
4	04I462	22/12/2004	*M*. *abscessus* subsp. *bolletii*	Profile IV	Lung	T (functional)	Wt	-	-	-	-	-	-
4	05I586	24/11/2005	*M*. *abscessus* subsp. *bolletii*	Profile IV	Lung	T (functional)	Wt	-	-	-	-	-	-
5	00I031	01/12/1995	*M*. *abscessus* subsp. *bolletii*	ND	Lung	T (functional)	Wt	0.12	16	>16	0.38	16	>256
6	14I016	17/01/2014	*M*. *abscessus* subsp. *bolletii*	ND	Lung	T (functional)	Wt	0.12	8	>16	<0.016	0.38	>256
7	07I004	03/04/2007	*M*. *abscessus* subsp. *bolletii*	ND	Skin	Gen truncated (Not functional)	Wt	-	-	-	-	-	-
8	05I284	15/07/2005	*M*. *abscessus* subsp. *massiliense*	ND	Skin	Gen truncated (Not functional)	Wt	-	-	-	-	-	-
9	08I420	31/10/2008	*M*. *abscessus* subsp. *abscessus*	ND	Lung	T (functional)	Wt	0.12	16	>16	0.25	>256	>256
10	10I050	18/02/2010	*M*. *abscessus* subsp. *abscessus*	ND	Lung	T (functional)	Wt	-	-	-	-	-	-
11	06I323	20/08/2006	*M*. *abscessus* subsp. *abscessus*	ND	Lung	T (functional)	Wt	-	-	-	-	-	-
12	09I456	04/12/2009	*M*. *abscessus* subsp. *abscessus*	ND	Lung	T (functional)	Wt	-	-	-	-	-	-
13	08I004	28/02/2008	*M*. *abscessus* subsp. *abscessus*	ND	Skin	T (functional)	Wt	0.25	8	>16	0.125	8	>256
14	01I019	03/10/1997	*M*. *abscessus* subsp. *abscessus*	ND	Lung	T (functional)	Wt	0.12	>16	>16	0.125	192	>256
15	14I019	23/01/2014	*M*. *abscessus* subsp. *abscessus*	ND	Lung	C (Not functional)	Wt	0.06	0.12	<2	<0.016	<0.016	<1
16	14I069	13/03/2014	*M*. *abscessus* subsp. *abscessus*	ND	Skin	C (Not functional)	Wt	-	-	-	-	-	-

^a^ ND, Not determined

^b^ nt, Nucleotide

^c^ Wt, Wild type

^d^ D, days

^e^ MIC values in μg/mL

^f^ MIC ranges for microdilution broth: ≤ 2, Susceptible; ≥ 8, Resistant

^g^ MIC ranges for E-test: ≤ 1, Susceptible; ≥ 4, Resistant

^h^ The hyphen indicates that phenotypic techniques were not performed due to strain non-recovery.

^i^ Double peak in DNA sequence

### Genetic basis of resistance to clarithromycin in *M*. *abscessus* complex

Table 1 also shows *rrl* and *erm(41)* results and the type of resistance in initial isolates. No mutations were observed in the *rrl* gene in any of these isolates. For the *erm(41)* T28 polymorphism, we identified 8 *M*. *abscessus* subsp. *abscessus* and 3 *M*. *abscessus* subsp. *bolletii*. For the *erm(41)* C28 polymorphism, we identified 3 *M*. *abscessus* subsp. *abscessus*. We found an *erm(41)* truncated gene in 1 *M*. *abscessus* subsp. *massiliense* and in 1 *M*. *abscessus* subsp. *bolletii*, in each case with two deletions at positions 64 and 65 and a final deletion of 276 bp, as described in the literature for *M*. *abscessus* subsp. *massiliense* [16].

For the first time, to our knowledge, in this study was found an *erm(41)* gene truncated in 1 *M*. *abscessus* subsp *bolletii*. Data of the similarity of the *rpoB*, *secA1*, *sodA* and *hsp65* gene sequences of this strain and the reference strains of *M*. *abscessus* complex is shown in the Table 2.

**Table 2:** Comparison between *Mycobacterium abscessus* subsp. *bolletii* 07I004 strain and *Mycobacterium* abscessus complex reference strains.

		Percentage of identity			
Strain	Reference Strains	*rpoB*	*hsp65*	*secA1*	*sodA*
7I004 *M*. *abscessus* subsp. *bolletii*	*M*. *abscessus* subsp. *abscessus* ATCC 19977T	95.75	98.6	98.02	98.64
	*M*. *abscessus* subsp. *bolletii* CCGU 50184 ^b^ and CIP 108541 ^c^	**99.45**	**100**	**100**	**99.79**
	*M*. *abscessus* subsp. *massiliense* CCUG48898	98.27	99.3	98.71	98.46

^a^ Values shown in bold indicate the highest percentage of identity

^b^ *M*. *abscessus* subsp. *bolletii* CCGU 50184 used for *rpoB* and *secA1* genes.

^c^ *M*. *abscessus* subsp. *bolletii* CIP 108541 used for *hsp65* and *sodA* genes.

### Clarithromycin susceptibility testing

We recovered 9 strains of the *M*. *abscessus* group for phenotypic analysis. E-test and microdilution methods were performed with recovered strains to compare the two methods and relate the *in vitro* MIC results with our genotypic analysis. Table 1 shows clarithromycin MIC for the E-test and microdilution. All strains were clarithromycin-susceptible with both techniques at 3 days. Five out of nine strains were resistant at 7 days for both methods and two strains were susceptible. One strain was resistant for E-test and susceptible for microdilution, the remaining strain was susceptible for E-test and resistant for microdilution (Table 1). At day 14, 7/9 strains were resistant with both methods. In both methods, susceptible and resistant strains agreed at day 14.

### Patient follow-up

Table 1 also describes the follow-up results for 4 patients: *M*. *abscessus* subsp. *abscessus* strains obtained from 3 patients (Patients 1–3) showed the acquisition of a point mutation in the *rrl* gene. Patient 1, *erm(41)* C28 sequevar, showed an acquired resistance at position A2058G. Patient 3, *erm(41)* T28 sequevar, showed an acquired resistance at position A2059G. The *erm(41)* T28 sequevar from patient 2 showed a double peak at position A2057G/A in the DNA sequence, indicating a new selected mutation for clarithromycin constitutive resistance. In contrast, the *M*. *abscessus* subsp. *bolletii* strain from patient 4 showed no acquired mutation in the *rrl* gene.

All the strains studied in the follow-up were identical to the initial isolates for each patient. Each patient had a different strain. Table 1 also shows the profile of the variable-number of tandem-repeat assay.

## Discussion

Clarithromycin is a key agent in the treatment of *M*. *abscessus* complex infections [9]. The main cause of treatment failure is inducible resistance [8,21]. Significant differences in the *erm(41)* gene associated with inducible resistance are found among the *M*. *abscessus* complex [15,22], suggesting that an accurate *erm(41*) allele identification is important to predict treatment outcomes. As the three *M*. *abscessus* complex subspecies are closely related and show horizontal gene transfer [11,23], more than one housekeeping gene is needed for subspecies identification [13]. Nevertheless, the genes to be used are not defined [24]. This work aimed to characterize the molecular mechanisms of clarithromycin resistance in the *M*. *abscessus* complex and to verify the relationship between these mechanisms and the clarithromycin susceptibility test.

In contrast with some authors [12,13] and in agreement with Nie *et al*. [25], we found each gene (*rpoB*, *secA1*, *sodA* and *hsp65*) was able to classify *M*. *abscessus* subspecies, with the exception of one strain, which was identified as *M*. *abscessus* subsp. *abscessus* by *rpoB*, *sodA* and *hsp65*, and *M*. *abscessus* subsp. *bolletii* by *secA1* gene. In contrast with our results, Tan *et al*. [26] were able to correctly classify all their strains by sequencing the *secA1* gene. *M*. *abscessus* subsp. *abscessus* was the most predominant subspecies of the complex (68.8%), in keeping with findings in the literature [12,21,27], where it ranges from 51.2% to 78.5%. Next in predominance was *M*. *abscessus* subsp. *bolletii* (25.0%), in contrast with many papers where the second most frequent subspecies is *M*. *abscessus* subsp. *massiliense* [16,28].

Regarding genetic resistance mechanisms, *rrl* gene sequencing in the initial isolates showed an absence of mutations. This is in contrast with previous reports [21,29], where the presence of constitutive clarithromycin resistance ranges from 2.7% to 28.6%. In agreement with the literature, however, we observed inducible resistance due to nucleotide T28 of *erm(41)* in 68.8% (11/16) of *M*. *abscessus* complex isolates [8,21].

As previously reported, *erm(41)* is a subspecies-specific gene in the *M*. *abscessus* group [7,16]. We found only one discrepancy with respect to the published criteria [7,17], attributed to an *M*. *abscessus* subsp. *bolletii* strain with a truncated *erm(41)* gene. To our knowledge, this is the first description of *M*. *abscessus* subsp. *bolletii* with a non-functional truncated *erm(41)* gene. The strain (07I004) matched *M*. *abscessus* subsp. *bolletii* reference strains CCUG 50184 and CIP108541 for the four housekeeping genes studied but not for the *erm(41)* gene. This finding supports other reports suggesting that gene transfer and recombination occurs between subspecies [11,28]. Several cases of *M*. *abscessus* subsp. *massiliense* with the functional *erm(41)* gene have been reported [30]. Our data thus support the notion that *erm(41)* is not a subspecies-specific gene, and that *erm(41)* gene sequencing should not be used as the only technique to classify *M*. *abscessus* complex subspecies.

The data produced by our study revealed the acquisition of constitutive clarithromycin resistance. Constitutive resistance to clarithromycin due to mutations in *rrl* was selected in both *erm(41)* C28 (as shown in Patient 1) [7] and *erm(41)* T28 strains (as shown in Patients 2 and 3) [21]. With the advantage of having the initial *rrl* wild-type strains, our results support the idea, in opposition to Bastian *et al*. [7], that selection of *rrl* mutants is similar in T28 and C28 strains.

We described the acquisition of a 2057 point mutation in a T28 *M*. *abscessus* subsp. *abscessus* strain with an initial *rrl* wild-type isolate. A double peak in the 2057 DNA sequence was observed, which suggests the coexistence of two mixed DNA populations (an *rrl* wild-type and a 2057 mutated population), since these mycobacteria possess only a single copy of the *rrl* gene in their genome [5]. The amplification and DNA sequencing was repeated three independent times to discard amplification or sequencing errors. Further work should include cloning to determine whether two variants exist in this strain.

It is important to emphasize that mutations at position 2057 of *rrl* in other microorganisms are related to medium-low levels of clarithromycin resistance *in vitro* [31], as seen in this study. Vester *et al*. [31] also reported that mutations at positions 2057 and 2611 of the *rrl* gene caused resistance to macrolides and ketolides due to their proximity to the action centre. Mutations at these positions obstruct the link between the antibiotic and its target, and cause low-level resistance to 14-membered-ring macrolides and no resistance to 16-membered-ring macrolides. In contrast, recently, Luo *et al*. [32] reported a 2057 point mutation in a *M*. *abscessus* subsp. *abscessus* which showed a high level resistance (MIC >256 μg/mL).

In our study we found complete agreement between *erm(41)*, *rrl* sequencing and clarithromycin susceptibility testing. After a 3-day culture, all strains were susceptible as was observed with both methods (E-test and microdilution). Following a 7-day culture, 85% (6/7) of inducible resistance [*erm(41)* T28 sequevar] was detected, and after a 14-day culture, we detected all inducible resistance strains. Although we did not find significant differences between the two methods, one strain with the E-test and one strain with the microdilution method took 14 days to reveal resistance. This is in contrast with other authors who report differences between E-test and microdilution performance [33]. Although the microdilution is the standard method according to the CLSI [9], in our study, the E-test showed the same results as the microdilution, and MIC reading at early stages was easier.

We would like to emphasize that one of the limitations of our study is the relatively small amount of isolates included. This is important to consider when drowning conclusions on epidemiology, but it does not invalidate the molecular observations.

## Conclusion

In conclusion, our findings identified one strain of *M*. *abscessus* subsp. *bolletii* with a truncated and non-functional *erm(41)* gene. Caution is needed if *erm(41)* gene sequencing is the only technique used for subspecies identification. Although clarithromycin constitutive resistance is not detected in the initial isolates, it can be acquired in T28 and C28 *erm(41)* strains of the *M*. *abscessus* complex. Finally, the acquisition of a mutation at position 2057 in the *rrl* gene confers medium-low level resistance to clarithromycin.

## References

[1] Griffith DE, Girard WM, Wallace RJ. Clinical features of pulmonary disease caused by rapidly growing mycobacteria. An analysis of 154 patients. Am Rev Respir Dis. 1993;147: 1271–8. 10.1164/ajrccm/147.5.1271 8484642

[2] Wallace RJ, Swenson JM, Silcox VA, Good RC, Tschen JA, Stone MS. Spectrum of disease due to rapidly growing mycobacteria. Rev Infect Dis. 5: 657–79. 6353528 10.1093/clinids/5.4.657

[3] Nessar R, Cambau E, Reyrat JM, Murray A, Gicquel B. *Mycobacterium abscessus*: a new antibiotic nightmare. J Antimicrob Chemother. 2012;67: 810–8. 10.1093/jac/dkr578 22290346

[4] Lee SH, Yoo HK, Kim SH, Koh W-J, Kim CK, Park YK, et al The drug resistance profile of *Mycobacterium abscessus* group strains from Korea. Ann Lab Med. 2014;34: 31–7. 10.3343/alm.2014.34.1.31 24422193 PMC3885770

[5] Griffith DE, Aksamit T, Brown-Elliott B a, Catanzaro A, Daley C, Gordin F, et al An official ATS/IDSA statement: diagnosis, treatment, and prevention of nontuberculous mycobacterial diseases. Am J Respir Crit Care Med. 2007;175: 367–416. 10.1164/rccm.200604-571ST 17277290

[6] Brown-Elliot B A, Nash K A, Wallace R J.Antimicrobial Susceptibility Testing, Drug Resistance Mechanisms, and Therapy of Infections with Nontubercuolous Mycobacteria. Clin Microbiol Rev. 2012;25: 545–582. 10.1128/CMR.05030-11 22763637 PMC3416486

[7] Bastian S, Veziris N, Roux A-L, Brossier F, Gaillard J-L, Jarlier V, et al Assessment of clarithromycin susceptibility in strains belonging to the *Mycobacterium abscessus* group by *erm(41*) and *rrl* sequencing. Antimicrob Agents Chemother. 2011;55: 775–81. 10.1128/AAC.00861-10 21135185 PMC3028756

[8] Nash K a, Brown-Elliott B a, Wallace RJ. A novel gene, *erm(41)*, confers inducible macrolide resistance to clinical isolates of *Mycobacterium abscessus* but is absent from *Mycobacterium chelonae* . Antimicrob Agents Chemother. 2009;53: 1367–76. 10.1128/AAC.01275-08 19171799 PMC2663066

[9] Clinical & Laboratory Standards Institute, 2011. M24-A2: Susceptibility Testing of Mycobacteria, Nocardiae, and Other Aerobic Actinomycetes; Approved Standard Second Edition—M24A2. 31339680

[10] Leao SC, Tortoli E, Euzéby JP, Garcia MJ. Proposal that *Mycobacterium massiliense* and *Mycobacterium bolletii* be united and reclassified as *Mycobacterium abscessus* subsp. *bolletii comb*. *nov*., designation of *Mycobacterium abscessus* subsp. *abscessus* subsp. nov. and emended description of Mycobacteri. Int J Syst Evol Microbiol. 2011;61: 2311–3. 10.1099/ijs.0.023770-0 21037035

[11] Sassi M, Drancourt M. Genome analysis reveals three genomospecies in *Mycobacterium abscessus* . BMC Genomics. 2014;15: 359 10.1186/1471-2164-15-359 24886480 PMC4035080

[12] Zelazny AM, Root JM, Shea YR, Colombo RE, Shamputa IC, Stock F, et al Cohort study of molecular identification and typing of *Mycobacterium abscessus*, *Mycobacterium massiliense*, *and Mycobacterium bolletii* . J Clin Microbiol. 2009;47: 1985–95. 10.1128/JCM.01688-08 19420162 PMC2708513

[13] Macheras E, Roux A-L, Ripoll F, Sivadon-Tardy V, Gutierrez C, Gaillard J-L, et al Inaccuracy of single-target sequencing for discriminating species of the *Mycobacterium abscessus* group. J Clin Microbiol. 2009;47: 2596–600. 10.1128/JCM.00037-09 19515839 PMC2725694

[14] Fangous M-S, Mougari F, Gouriou S, Calvez E, Raskine L, Cambau E, et al A classification algorithm for subspecies identification within the *Mycobacterium abscessus* species, based on Matrix-Assisted Laser-Desorption/Ionization Time-of-Flight Mass Spectrometry. J Clin Microbiol. 2014; 10.1128/JCM.00788-14 25009048 PMC4313163

[15] Brown-Elliott B A, Vasireddy S, Vasireddy R, Iakhiaeva E, Howard ST, Nash K, et al Utility of sequencing the *erm(41)* gene in isolates of *Mycobacterium abscessus* subsp. *abscessus* with low and intermediate clarithromycin MICs. J Clin Microbiol. 2015;53: 1211–5. 10.1128/JCM.02950-14 25653399 PMC4365201

[16] Kim H-Y, Kim BJB-J, Kook Y-HY, Yun Y-J, Shin JH. *Mycobacterium massiliense* is differentiated from *Mycobacterium abscessus* and *Mycobacterium bolletii* by erythromycin ribosome methyltransferase gene *(erm)* and clarithromycin susceptibility patterns. Microbiol Immunol. 2010;54: 347–53. 10.1111/j.1348-0421.2010.00221.x 20536733

[17] Yoshida S, Tsuyuguchi K, Suzuki K, Tomita M, Okada M, Shimada R, et al Rapid identification of strains belonging to the *Mycobacterium abscessus* group through *erm(41)* gene pyrosequencing. Diagn Microbiol Infect Dis. Elsevier Inc.; 2014; 1–6. 10.1016/j.diagmicrobio.2014.04.001 24809859

[18] Zhang ZQ, Ishaque M. Evaluation of methods for isolation of DNA from slowly and rapidly growing mycobacteria. Int J Lepr Other Mycobact Dis. 1997:65:469–76. 9465157

[19] Biehle JONR, Cavalieri SJ, Saubolle MA. Evaluation of Etest for susceptibility testing of rapidly growing mycobacteria. These include: Evaluation of Etest for Susceptibility Testing of Rapidly Growing Mycobacteria. 1995;33. 10.1128/jcm.33.7.1760-1764.1995 PMC228264 7665643

[20] Wong YL, Ong CS, Ngeow YF. Molecular typing of *Mycobacterium abscessus* based on tandem-repeat polymorphism. J Clin Microbiol. 2012;50: 3084–8. 10.1128/JCM.00753-12 22760048 PMC3421802

[21] Lee S-H, Yoo HK, Kim SH, Koh W-J, Kim CK, Park YK, et al Detection and Assessment of Clarithromycin Inducible Resistant Strains Among Korean *Mycobacterium abscessus* Clinical Strains: PCR Methods. J Clin Lab Anal. 2014;6: 1–6. 10.1002/jcla.21702 PMC6807423 24652818

[22] Koh W-J, Jeon K, Lee NY, Kim B-J, Kook Y-H, Lee S-H, et al Clinical significance of differentiation of *Mycobacterium massiliense* from *Mycobacterium abscessus* . Am J Respir Crit Care Med. 2011;183: 405–10. 10.1164/rccm.201003-0395OC 20833823

[23] Choo SW, Wee WY, Ngeow YF, Mitchell W, Tan JL, Wong GJ, et al Genomic reconnaissance of clinical isolates of emerging human pathogen *Mycobacterium abscessus* reveals high evolutionary potential. Sci Rep. 2014;4: 4061 10.1038/srep04061 24515248 PMC3920477

[24] Blauwendraat C, Dixon GLJ, Hartley JC, Foweraker J, Harris K A. The use of a two-gene sequencing approach to accurately distinguish between the species within the *Mycobacterium abscessus* complex and *Mycobacterium chelonae* . Eur J Clin Microbiol Infect Dis. 2012;31: 1847–53. 10.1007/s10096-011-1510-9 22222989

[25] Nie W, Duan H, Huang H, Lu Y, Bi D, Chu N. Species identification using *rpoB* and *hsp65* and susceptibility testing to eight antibiotics of *Mycobacterium abscessus* subsp. *abscessus* and *Mycobacterium abscessus* subsp. *bolletii* . Int J Infect Dis. 2014; 10.1016/j.ijid.2014.02.014 24932856

[26] Tan JL, Khang TF, Ngeow YF, Choo SW. A phylogenomic approach to bacterial subspecies classification: proof of concept in *Mycobacterium abscessus* . BMC Genomics. 2013;14: 879 10.1186/1471-2164-14-879 24330254 PMC3878664

[27] Kim H-Y, Kook Y, Yun Y-J, Park CG, Lee NY, Shim TS, et al Proportions of *Mycobacterium massiliense* and *Mycobacterium bolletii* strains among Korean *Mycobacterium chelonae-Mycobacterium abscessus* group isolates. J Clin Microbiol. 2008;46: 3384–90. 10.1128/JCM.00319-08 18753344 PMC2566064

[28] Cho Y-J, Yi H, Chun J, Cho S-N, Daley CL, Koh W-J, et al The genome sequence of “*Mycobacterium massiliense*” strain CIP 108297 suggests the independent taxonomic status of the *Mycobacterium abscessus* complex at the subspecies level. PLoS One. 2013;8: e81560 10.1371/journal.pone.0081560 24312320 PMC3842311

[29] Yoshida S, Tsuyuguchi K, Suzuki K, Tomita M, Okada M, Hayashi S, et al Further isolation of *Mycobacterium abscessus* subsp. *abscessus* and subsp. *bolletii* in different regions of Japan and susceptibility of these isolates to antimicrobial agents. Int J Antimicrob Agents. Elsevier B.V.; 2013;42: 226–31. 10.1016/j.ijantimicag.2013.04.029 23850022

[30] Gray TJ, Kong F, Jelfs P, Sintchenko V, Chen SC. Improved Identification of Rapidly Growing Mycobacteria by a 16S-23S Internal Transcribed Spacer Region PCR and Capillary Gel Electrophoresis. PLoS One. 2014;9: e102290 10.1371/journal.pone.0102290 25013955 PMC4094492

[31] Vester B, Douthwaite S. Macrolide Resistance Conferred by Base Substitutions in 23S rRNA MINIREVIEW Antimicrob Agents Chemother. 2001;45:1–12. 10.1128/AAC.45.1.1-12.2001 PMC90232 11120937

[32] Luo RF, Curry C, Taylor N, Budvytiene I, Banaei N. Rapid Detection of Acquired and Inducible Clarithromycin Resistance in *Mycobacterium abscessus* group by a Simple Real-Time PCR Assay. J Clin Microbiol. 2015; 1–12. 10.1128/JCM.00132-15 25903572 PMC4473208

[33] Woods GL, Bergmann JS, Witebsky FG, Fahle GA, Boulet B, Plaunt M, et al Multisite Reproducibility of Etest for Susceptibility Testing of *Mycobacterium abscessus*, *Mycobacterium chelonae*, and *Mycobacterium fortuitum* . J Clin Microbiol. 2000;38:656–61. 10655363 10.1128/jcm.38.2.656-661.2000 PMC86169

